# Dose-adjusted EPOCH plus rituximab improves the clinical outcome of young patients affected by double expressor diffuse large B-cell lymphoma

**DOI:** 10.1038/s41375-018-0320-9

**Published:** 2019-01-10

**Authors:** A. Dodero, A. Guidetti, A. Tucci, F. Barretta, M. Novo, L. Devizzi, A. Re, A. Passi, A. Pellegrinelli, G. Pruneri, R. Miceli, A. Testi, M. Pennisi, M. C. Di Chio, P. Matteucci, C. Carniti, F. Facchetti, G. Rossi, P. Corradini

**Affiliations:** 1 Department of Hematology, Fondazione IRCCS Istituto Nazionale dei Tumori, Milano, Italy; 2 Department of Oncology and Hemato-Oncology, University of Milano, Milano, Italy; 3 Department of Hematology, Azienda Ospedaliera Spedali Civili di Brescia, Brescia, Italy; 4 Department of Clinical Epidemiology and Trial Organization, Fondazione IRCCS Istituto Nazionale dei Tumori, Milano, Italy; 5 Department of Hematology, Azienda Ospedaliero Universitaria Citta’ della Salute e della Scienza di Torino, Torino, Italy; 6 Department of Pathology, Fondazione IRCCS Istituto Nazionale dei Tumori, Milano, Italy; 7 Department of Pathology, Azienda Ospedaliera Spedali Civili di Brescia, Brescia, Italy

**Keywords:** Drug development, Combination drug therapy

## To the Editor:

Diffuse large B-cell lymphoma (DLBCL) is a heterogeneous disease. Patients carrying the double expression of MYC and BCL2 (double expressor, DE) with or without concomitant translocations of MYC and BCL2, and/or BCL6 genes have a dismal prognosis. In patients affected by DE DLBCL without translocations, the 5-year overall survival (OS) is ~40% with R-CHOP. In high grade B-cell lymphomas [i.e., double hit (DH) or triple hit (TH) lymphomas], the median OS is ~12 months [1–6]. Therefore, no standard therapy for these disease entities exists, and their optimal treatment represents an urgent unmet clinical need.

At our Institution, the DA-EPOCH-R has been adopted for all DE DLBCL patients since 2013. The present study compared the outcome of patients treated in a similar period at three different Italian institutions with DA-EPOCH-R and R-CHOP. We estimated the propensity score (PS) as a balancing score to account for the biases consistent with a non-random treatment assignment. The treatment effect in the multivariable Cox models was estimated using an inverse-probability-of-treatment-weight (IPTW) based on PS [7].

Diagnosis was performed according to the WHO classification and was reviewed by two expert hematopathologists [patients with primary mediastinal and human immunodeficiency virus-associated lymphomas or central nervous system (CNS) disease were excluded]. Immunohistochemistry analysis and FISH were performed in all patients and are detailed in Supplementary Appendix. The cut-off levels for positivity for MYC and BCL2 were ≥40% and ≥50%, respectively [8]. In this analysis, we included stages II–IV or stage I disease with an International Prognostic Index (IPI) score ≥ 1 or bulky disease. The Ethical Committees of participating centers approved the study (INT55/17). Written informed consent was obtained from all patients.

The DA-EPOCH-R regimen was administered as previously described every 21 days for 6 cycles and dose-adjustment was based on blood counts between cycles [9]. R-CHOP was administered every 21 days. CNS prophylaxis with lumbar puncture or intravenous methotrexate was administered according to institutional guidelines. Disease assessment during the study was described in Supplementary Appendix.

The primary objective of the study was to compare the 2-year PFS and OS in the DA-EPOCH-R and R-CHOP cohorts. Secondary objectives included comparisons of survival according to different prognostic factors (age, stage, IPI, cell of origin and cytogenetic characterization). Statistical Methods are summarized in Supplementary Appendix.

A total of 114 consecutive patients were identified for the study. Table 1 summarizes the main clinical and biological characteristics of the patients. Fifty-one patients received DA-EPOCH-R between October 2013 and October 2017 while 63 patients were treated with R-CHOP between October 2009 and October 2017. Cohorts were well balanced across all covariates included in the PS model after IPTW adjustment as indicated by the standardized mean difference (SMD) values calculated after weighting (<0.1). The overall response rates were 80% [CR, *n* = 37 (73%); PR, *n* = 4], and 76% [CR, *n* = 44 (71%); PR, *n* = 4] following DA-EPOCH-R and R-CHOP, respectively.

**Table 1:** Clinical and biological characteristics

Variable	ALL	DA-EPOCH-R	R-CHOP	SMD before/after
	*N* = 114	*N* = 51	*N* = 63	
Age				
Median	62 years	58 years	65 years	0.296/<0.001
Range	29–81	29–79	36–81	
Age > 65 years	43 (38%)	14 (27%)	29 (46%)	
Sex				
Male	70 (62%)	32 (63%)	38 (60%)	0.049/0.018
Female	44 (38%)	19 (37%)	25 (40%)	
Histology				
DLBCL	106 (93%)	47(92%)	59 (94%)	
Transformed	8 (7%)	4 (8%)	4 (6%)	—
Stage				
I–II	28 (25%)	8 (16%)	20 (32%)	0.850/0.079
III–IV	86 (75%)	43 (84%)	43 (68%)	
IPI				
1–2	62 (54%)	24 (47%)	38 (60%)	0.332/0.101
3–5	52 (46%)	27 (53%)	25 (40%)	
BM involvement				
Yes	22 (19%)	16 (32%)	6 (10%)	0.552/0.421
No	92 (81%)	35 (68%)	57 (90%)	
Extranodal involvement^a^				
Yes	69 (60%)	46 (90%)	23 (36%)	—
No	45 (40%)	5 (10%)	40 (64%)	
CNS involvement				
Leptomeningeal	3 (3%)	2 (4%)	1 (1%)	—
Cytogenetic abnormalities				
DE-only	58 (51%)	18 (35%)	40 (63%)	0.749/0.119
DE with SH	29 (25%)	16 (31%)	13 (21%)	
DE with DH/TH	10 (9%)	8 (16%)	2 (3%)	
DE with atypical DH	15 (13%)	9 (18%)	6 (10%)	
Missing	2 (2%)	0 (0%)	2 (3%)	
FISH rearrangements				
MYC R	7 (6%)	5 (10%)		—
BCL2 R	8 (7%)	5 (10%)	2 (3%)	
BCL6 R	14 (12%)	6 (12%)	3 (5%)	
MYC R/BCL2 R	5 (4%)	4 (7%)	8 (13%)	
MYC R/BCL6 R	2 (2%)	2 (4%)	1 (2%)	
MYC R/BCL2 R/BCL6 R	3 (3%)	2 (4%)	0 (0%)	
Cell of origin (Hans)				0.334/0.049
GCB	42 (37%)	22 (43%)	20 (31%)	
Non GCB	57 (50%)	25 (49%)	32 (51%)	
Not evaluable	15 (13%)	4 (8%)	11 (18%)	

*SMD* Standardized mean difference calculated before and after weighting, *DLBCL* diffuse large B-cell lymphomas, *BM* bone marrow, *FISH* fluorescence in situ hybridization, *DE* double expressor, *SH* single-hit, *DH* double hit, *TH* triple hit, *R* rearrangements, *GCB* germinal center B-cell lymphomas

^a^At least one extranodal involvement

Patients in the R-CHOP group received less CNS prophylaxis compared to those treated with DA-EPOCH-R (30% vs 96%). In particular, since 2016 patients in the DA-EPOCH-R group [23 of 51 (45%)] received four lumbar punctures with methotrexate, cytarabine, and dexamethasone followed by intravenous Methotrexate (3.5 g/ms, for 2 courses) whereas all other patients received triple intrathecal prophylaxis only. CNS relapses following DA-EPOCH-R and R-CHOP were observed in one and three patients, respectively. Consolidative radiotherapy was administered in 21% and 35% of the patients in the DA-EPOCH-R and R-CHOP cohorts, respectively. Death occurred in 6 (12%) patients receiving DA-EPOCH-R [PD (*n* = 4), pneumonia (*n* = 1), suicide (*n* = 1)], and in 22 (35%) R-CHOP patients [PD (*n* = 20), toxicity (*n* = 2)], respectively.

DA-EPOCH-R dose escalation was feasible in most patients aged ≤65 years with 73% of the patients being escalated to the 3rd dose level or above (Supplementary Figure 1). In contrast, the majority of the elderly patients (≥65 years) received therapy at level 1 (11 out of 14, 78%) due to comorbidities and toxicities. Supplementary Table 1 lists the primary toxicities observed during DA-EPOCH-R. Therapy discontinuation due to adverse events occurred in two patients per arm [DA-EPOCH-R: pneumonia (Grade 5) and infection (alive in CR); R-CHOP: toxicity (Grade 5, *n* = 2)].

**Fig. 1:**
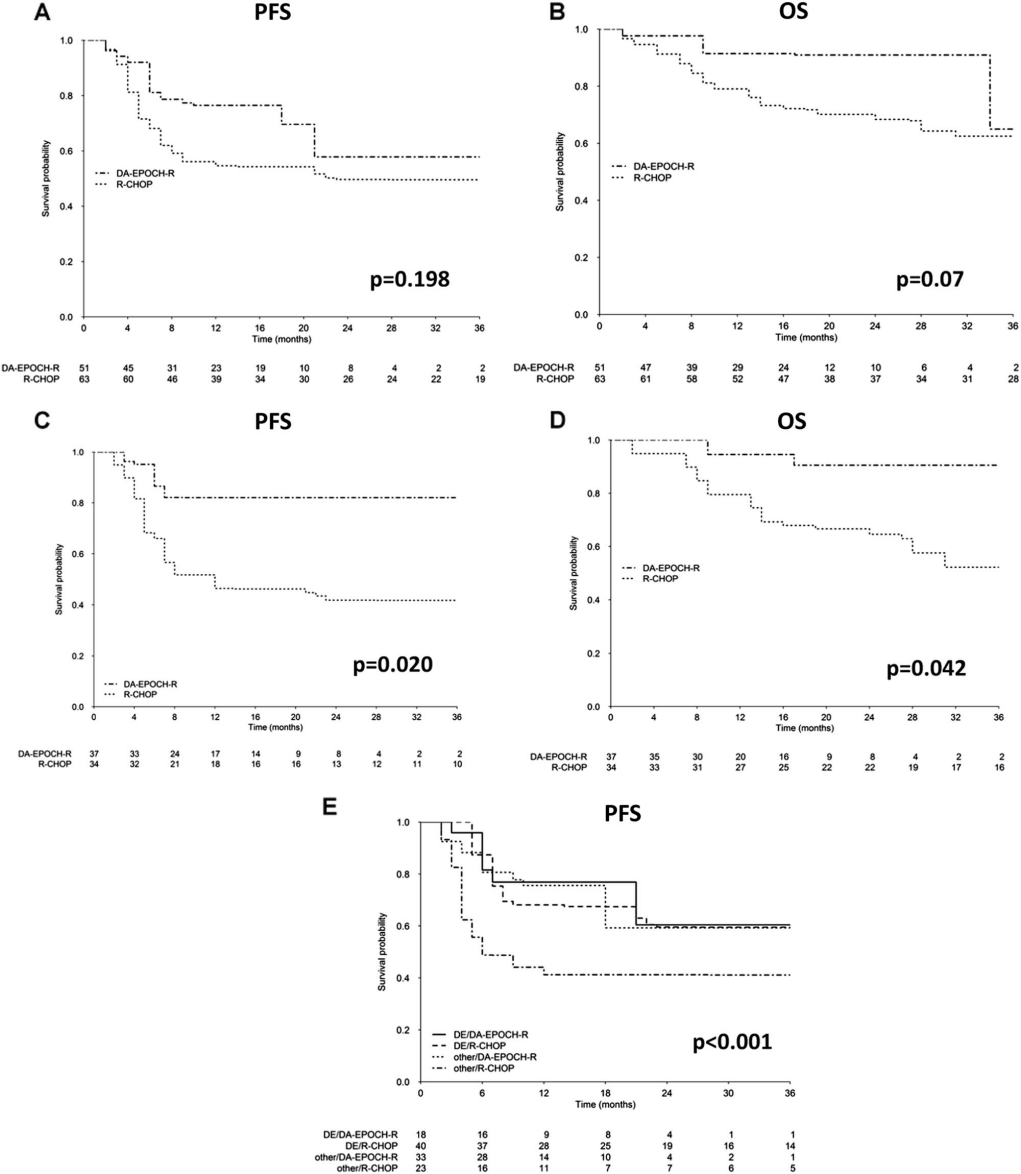
Weighted Kaplan–Meier curves of progression-free survival (**a**) and overall survival (**b**) in all patients according to treatment (DA-EPOCH-R or R-CHOP); Weighted Kaplan–Meier curves of progression-free survival (**c**) and overall survival (**d**) according to treatment in patients younger than 65 years. Weighted Kaplan–Meier estimates of progression-free survival according to biology (DE patients with cytogenetic alteration such as SH, DH/TH, or atypical DH defined as “other” vs patients without any abnormalities defined as “DE-only”) and treatment (**e**)

Median follow-ups were 20 and 49 months in the DA-EPOCH-R and R-CHOP group, respectively. The 2-year PFS and OS, before IPTW adjustment, were 62% (95% CI: 45–84%) and 85% (95% CI: 74–98%) following DA-EPOCH-R and 54% (95% CI: 43–69%) and 70% (95% CI: 60–83%) following R-CHOP, respectively. After weighting no significant statistical differences in the 2-year PFS (57% vs 51%, *p* = 0.198) or OS (90% vs 67%, *p* = 0.07) were observed following DA-EPOCH-R or R-CHOP, respectively (Fig. 1a, b).

We then used an IPTW based on PS to estimate the potential impact of the two regimens on age (≤65 vs >65), disease stage (limited vs advanced), IPI [low risk (IPI 1–2) vs high risk (IPI 3–5)], non-Germinal Center B-cell like subgroup. In this analysis, age emerged as a clinically relevant variable. In fact, patients aged <65 years treated with DA-EPOCH-R exhibited a better PFS [82% (95% CI: 66–100%) vs 43% (95% CI: 26–73%), *p* = 0.020] and a better OS [90% (95% CI: 74–100%) vs 62% (95% CI: 43–88%), *p* = 0.042] compared to those receiving R-CHOP (Fig. 1c, d). We did not observe any significant advantage for any other subgroup.

Analyses with IPTW adjustment were also performed on the entire cohort according to treatment (DA-EPOCH-R vs R-CHOP) and absence or presence of genetic lesions (DE-only including patients lacking cytogenetic alterations, vs “other” including single-hit, DH/TH or atypical DH patients [10]). The subgroup of patients with any type of cytogenetic abnormality treated with R-CHOP, had a worse 2-year PFS compared to the other subgroups (41% vs 59%, *p* < 0.001) (Fig. 1e).

Survival analysis of the entire study population revealed similar OS and PFS for R-CHOP and DA-EPOCH-R patients. In contrast, patients younger than 65 years achieved a 2-year PFS of 82%, which was significantly better than the PFS observed with R-CHOP. Most of the patients older than 65 years were treated with the first dose level compared to the group of younger patients who generally received level 3 or above (73% of cases) (Supplementary Figure 1). This suggests that the efficacy of the treatment in the younger population might be ascribed to the higher cumulative dose of chemotherapy received and not only to the continuous infusion of the drugs.

A possible counfounding factor in this study relates to CNS prophylaxis which varied over time. Biomarkers influencing CNS relapse risk, independently from clinical risk model (CNS-IPI), are in fact a recent acquisition. However, the observed rate of CNS relapse is in line with previous publications describing a risk of 13% and 9.7% at 2 years in patients affected by DH/TH and DE lymphomas, respectively [11, 12].

In this retrospective study, possible biases between R-CHOP and DA-EPOCH-R cohorts were balanced using stabilized IPTW based on the PS in all survival analyses. Our results indicate the potential role of DA-EPOCH-R in the treatment of DE DLBCL patients mainly because of three relevant observations: (1) treatment with DA-EPOCH-R compared to R-CHOP was associated with a significant improvement of PFS and OS in patients younger than 65 years; (2) all patients with genetic abnormalities (single translocations, atypical DH, and DH/TH) had better PFS with DA-EPOCH-R; and (3) intensification with DA-EPOCH-R was feasible and safe in young patients.

It is clear that the optimal chemo-immunotherapy for patients affected by DE DLBCL with or without gene rearrangements is still a matter of debate. However, the results observed with R-CHOP are considered unsatisfactory, and most published studies using more intensive regimens were retrospective and limited to the DH/TH subgroup [13, 14]. To our knowledge, this is the first report analyzing the survival of a consecutive cohort of genetically characterized DE patients treated with DA-EPOCH-R. In addition to DH/TH lymphomas, ~30–50% of patients in both cohorts exhibited single translocations or were atypical DH. We observed that DE patients carrying any genetic abnormality had a poor PFS when treated with R-CHOP while they experienced a significantly better outcome with DA-EPOCH-R. Separate survival analyses according to single cytogenetic alteration or combining age and cytogenetic alterations were not possible because of the limited number of patients in each subgroup.

It is true that the median follow-up for DA-EPOCH-R (20 months) cohort is shorter than R-CHOP (49 months), but recent studies showed that PFS at 24 months is a valuable surrogate end-point for OS in DLBCL patients [15]. The 2-year PFS following DA-EPOCH-R in younger patients is promising, but a prospective trial is required to confirm these findings.

In conclusion, the DA-EPOCH-R regimen is feasible in patients up to 79 years of age, but the advantage of using an intensive regimen is likely related to the dose escalation. The results of the present study suggest that intensive chemotherapy, such as DA-EPOCH-R, should be considered for patients with DE DLBCL aged less than 65 years.

## Supplementary information

Supplementary Appendix

Supplementary Table

Supplementary Figure 1
