# Supplementary material for: Dose-adjusted EPOCH plus rituximab improves the clinical outcome of young patients affected by double expressor diffuse large B-cell lymphoma
Source: Leukemia. 2019 Jan 10;33(4):1047–51. doi: 10.1038/s41375-018-0320-9 (PMC6756077; doi:10.1038/s41375-018-0320-9)
Supplement: Supplementary file 1 — Supplementary Appendix [file 41375_2018_320_MOESM1_ESM.docx]

**Patients and Methods:**

**Patients evaluation**

All patients underwent a computed tomography (CT) scan, positron emission tomography scan (PET), bone marrow biopsy and diagnostic lumbar puncture, including cytological and immunophenotype analyses, at baseline. An intermediate disease assessment using CT was performed after 3 or 4 DA-EPOCH-R or R-CHOP cycles. Patients who exhibited a less than partial response (PR) or progression of disease (PD) were shifted to second-line regimens according to institutional guidelines. Evaluation of final clinical response was performed at the end of cycle 6 using CT, PET and bone marrow biopsy, when the biopsy was positive at disease onset. Disease assessment was performed during follow-up at 3-month intervals for the first 2 years, every six months until the 5th year, and annually thereafter. Response evaluation was assessed using Lugano Revised Response Criteria^1^.

**Immunohistochemistry analysis and FISH**

Formalin-fixed, paraffin-embedded sections were deparaffinized and rehydrated. Representative tumour blocks were sectioned at 3-µm thickness. IHC was performed using the EnVision FLEX+, mouse, high pH (Link) method (Dako Denmark A/S Produktionsvej 42 DK-2600 Glostrup) and a Dako Autostainer Link48. Slides were stained with monoclonal antibodies against CD10, BCL2, BCL6, MUM1, MYC, and Ki67. All steps were performed at room temperature. 3-3’-Diaminobenzidine tetrahydrochloride (DAB) was incubated for 10 min as a chromogen, and Mayer’s haematoxylin was used for counterstaining for 10 min. The slides were dehydrated, cleared and mounted with coverslips. Cases were considered positive for MYC if ≥40% of tumour cells were stained with antibody. A cut-off level of 50% positive cells was used for BCL-2, according to a previous description^2^.

We used CD10, BCL-6 and MUM1 staining to divide all DLBCL cases into GCB or ABC subgroups according to the Hans algorithm^3^. The cut-off values for BCL-6, MUM-1 and CD10 were set at 30%, according to recent literature^2^. Fluorescence in situ hybridization (FISH) analyses for BCL2, BCL6 and MYC rearrangement were performed in all patients using “LSI BCL2 LSI BCL6 (ABR), C-MYC “break apart” probes (Vysis/Abbott Molecular) according to the manufacturer’s instructions. At least 100 nuclei were counted. Rearrangement was defined as the presence of break-apart signals in >15% of nuclei, and copy gain (CG) was defined as three or more signals in >30% of nuclei. DH and TH lymphomas were defined as concurrent rearrangement of MYC and BCL2 and/or BCL6.

Atypical DH lymphoma was defined as MYC CG without MYC rearrangement and with one or more of the following: BCL2 CG, BCL6 CG, BCL2 or BCL6 rearrangement; or MYC rearrangement without BCL2 or BCL6 rearrangement but with BCL2 CG and/or BCL6 CG^4^.

**Statistical Analyses**

PFS was defined as the time from diagnosis to recurrence or death, whichever occurred first. Time was censored at the latest follow-up for living patients who were relapse free. OS was defined as the time elapsed from diagnosis to death due to any cause. Time was censored at the latest follow-up for living patients.

We estimated a propensity score (PS) as a balancing score to account for the bias consistent with a non-random assignment of chemotherapy regimen (R-CHOP or DA-EPOCH-R)^5^ as detailed in Supplementary methods. Chemotherapy PS was estimated using a multivariable logistic model with binary response (R-CHOP or DA-EPOCH-R) and covariates, including age, stage (I-IV), IPI (1-5), genetics [DE-only, single rearrangement (SH), atypical DH, DH, TH, and missing values as a separate category], and GCB (GCB, not GCB, and missing values as a separate category). We used the method of stabilized weights^6^, which was proposed as a more advantageous analogue to one-to-one pair matching without replacement for the PS with a calliper to estimate an inverse-probability-of-treatment-weight (IPTW). PFS and OS curves were estimated using the weighted Kaplan-Meier method and compared using means of weighted log-rank tests.

IPTW multivariable Cox models were performed to assess the presence of interactions between chemotherapy and genetics (“DE-only” vs. “other”) in association with the endpoints by applying the IPTW. Standardized mean difference (SMD)^7^ was used to quantify differences in means (numerical variables) and proportions (categorical variables) between the two chemotherapy treatment groups, and SMDs ≥0.3 were considered indicative of a relevant between-group imbalance. SMD was calculated before and after IPTW adjustment.

The IPTW calculation and models and SMD analyses were performed on the entire series and different subgroups: DE genetics (DE-only, DH/TH, atypical DH, SH or missing), low IPI (1-2), high IPI (3-5), not GCB or missing, age below or equal 65 years, age over 65 years, limited stage (I-II), and advanced stage (III-IV). Propensity scores were re-estimated in each subgroup.

Patient age was modelled as a continuous variable using three-knot restricted cubic splines to obtain a flexible fit^8^. All other variables were modelled as categorical using dummy variables. Statistical analyses were performed using SAS^TM^ (SAS Institute, Cary, NC) and R software (R Foundation for Statistical Computing, Vienna, Austria).

**References**

1. Cheson BD, Fisher RI, Barrington SF, Cavalli F, Schwartz LH, Zucca E*, et al.* Recommendations for initial evaluation, staging, and response assessment of Hodgkin and non-Hodgkin lymphoma: the Lugano classification. *J Clin Oncol* 2014 Sep 20; **32**(27)**:** 3059-3068.

2. Swerdlow SH, Campo E, Pileri SA, Harris NL, Stein H, Siebert R*, et al.* The 2016 revision of the World Health Organization classification of lymphoid neoplasms. *Blood* 2016 May 19; **127**(20)**:** 2375-2390.

3. Hans CP, Weisenburger DD, Greiner TC, Gascoyne RD, Delabie J, Ott G*, et al.* Confirmation of the molecular classification of diffuse large B-cell lymphoma by immunohistochemistry using a tissue microarray. *Blood* 2004 Jan 1; **103**(1)**:** 275-282.

4. Li S, Seegmiller AC, Lin P, Wang XJ, Miranda RN, Bhagavathi S*, et al.* B-cell lymphomas with concurrent MYC and BCL2 abnormalities other than translocations behave similarly to MYC/BCL2 double-hit lymphomas. *Mod Pathol* 2015 Feb; **28**(2)**:** 208-217.

5. Rosenbaum PR, Rubin DB. Reducing bias in observational studies using subclassification on the propensity score. *J Am Stat Assoc* 1984; **79:** 516 - 524.

6. Austin PC, Stuart EA. Moving towards best practice when using inverse probability of treatment weighting (IPTW) using the propensity score to estimate causal treatment effects in observational studies. *Stat Med* 2015 Dec 10; **34**(28)**:** 3661-3679.

7. Flury BK, Reidwyl H. Standard distance in univariate and multivariate analysis. *The American Statistician* 1986; **40:** 249 - 251.

8. Durrleman S, Simon R. Flexible regression models with cubic splines. *Stat Med* 1989 May; **8**(5)**:** 551-561.
