# Supplementary material for: Dose-adjusted EPOCH plus rituximab improves the clinical outcome of young patients affected by double expressor diffuse large B-cell lymphoma
Source: Leukemia. 2019 Jan 10;33(4):1047–51. doi: 10.1038/s41375-018-0320-9 (PMC6756077; doi:10.1038/s41375-018-0320-9)
Supplement: Supplementary file 2 — Supplementary Table [file 41375_2018_320_MOESM2_ESM.docx]

**Supplementary Table.**

Primary toxicities observed during DA-EPOCH-R

| **Grade 3-4 Adverse Events – Total pts = 51** | **Pts (%)** |
| --- | --- |
| At least 1 adverse event (Grade 3-4) | 20 (39%) |
|  |  |
| Febrile Neutropaenia | 10 (20%) |
| Febrile Neutropaenia with Hospitalization | 4 (8%) |
| Peripheral neuropathy (including constipation) | 9 (18%) |
| Pneumonia | 4 (8%) |
| Deep Venous Thrombosis (including CVC-related) | 4 (8%) |
| Arrhythmia | 2 (2%) |
| Bowel perforation | 1 (4%) |
| Septic shock | 1 (2%) |
| Steroid-related arterious hypertension | 1 (2%) |
| Steroid-related osteoporosis | 1 (2%) |
