# Supplementary figures and images for: Dose-adjusted EPOCH plus rituximab improves the clinical outcome of young patients affected by double expressor diffuse large B-cell lymphoma
Source: Leukemia. 2019 Jan 10;33(4):1047–51. doi: 10.1038/s41375-018-0320-9 (PMC6756077; doi:10.1038/s41375-018-0320-9)

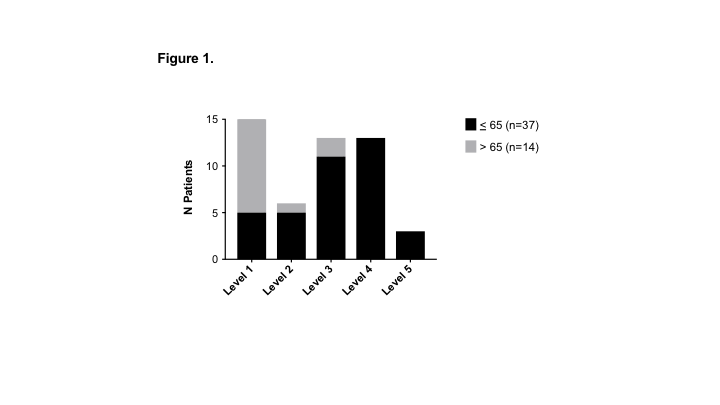

Supplement: Supplementary file 3 — Supplementary Figure 1 [file 41375_2018_320_MOESM3_ESM.tif]
